# Decomposition and oligomerization of 2,3-naphthyridine under high-pressure and high-temperature conditions

**DOI:** 10.1038/s41598-019-43868-2

**Published:** 2019-05-14

**Authors:** Ayako Shinozaki, Koichi Mimura, Tamihito Nishida

**Affiliations:** 1 Faculty of Science, Hokkaido University, N10 W8, Kita-ku, 060-0810 Sapporo, Hokkaido Japan; 2 Department of Earth and Planetary Sciences, Graduate School of Environmental Studies, Nagoya University, 464-8601 Nagoya, Japan

**Keywords:** Reaction mechanisms, Polymerization mechanisms

## Abstract

The chemical reaction of 2,3-naphthyridine, a nitrogen-containing aromatic compound, was investigated at pressures ranging from 0.5 to 1.5 GPa and temperatures from 473 to 573 K. A distinct decrease in the amount of residual 2,3-naphthyridine was observed in the samples recovered after reaction at ˃523 K at 0.5 and 1.0 GPa, and ˃548 K at 1.5 GPa. The formation of *o*-xylene and *o*-tolunitrile accompanied a decreasing N/C ratio of the reaction products, indicating decomposition of the aromatic ring and release of nitrogen. Precise analysis of the reaction products indicated the oligomerization of decomposed products with the residual 2,3-naphthyridine to form larger molecules up to 7mers. Nitrogen in the aromatic ring accelerated reactions to decompose the molecule and to oligomerize at lower temperatures than those typically reported for aromatic hydrocarbon oligomerization. The major reaction mechanism was similar between 0.5 and 1.5 GPa, although larger products preferentially formed in the samples at higher pressure.

## Introduction

Aromatic compounds are the most abundant organic materials in nature and are an important reservoir of light elements. They are found in extreme environments including interstellar clouds and planetary nebulas^1,2^, icy planets^3,4^, and carbonaceous meteorites^5,6^. The behavior of aromatic compounds in extreme conditions is an important subject in chemistry because of their distinctive planar molecular structure and delocalized π-electrons. Pressure-induced irreversible chemical reactions with amorphization of solid benzene and polycyclic aromatic hydrocarbons (PAHs) at room temperature have been reported^7–14^. Neighboring molecules in the solid crystal converge with increasing pressure and finally exceed an irreversible reaction threshold to form dimers and trimers^11–13,15^. In addition, a novel nanocarbon material called “carbon nanothread” was formed after the slow compression and decompression of benzene up to 20 GPa and exhibited a one-dimensional structure with sp^3^ bonding^16–18^. Applying higher pressure and temperature to aromatic compounds induces reactions that are more complex because decomposition occurs in addition to pressure-induced oligomerization/polymerization. Oligomerization of aromatic hydrocarbons was observed at 500–773 K at ambient pressure and at 3.5 GPa^19^ as well as at 773–973 K at 7 GPa^20,21^. During oligomerization, dehydrogenation and new C-C bond(s) formation maintain the aromatic rings^19^. Above a certain temperature, carbonization (graphitization) of the PAHs was observed and was considered to be the final stage of oligomerization^19,22,23^. Shock experiments on benzene, naphthalene, and other PAHs showed that amorphous carbon, hydrogen/hydrocarbon gases, and oligomers were formed with decreasing residual starting materials at shock pressures and temperatures above approximately 10 GPa and 800 K^24,25^.

A nitrogen atom can replace a CH group of an aromatic ring and maintain aromaticity, forming a heterocyclic aromatic compound, though the presence of nitrogen changes the electronic, physical, and chemical properties. A theoretical study suggested that dimerization via Diels-Alder reaction became slightly easier for nitrogen-containing aromatic compounds^26^. For nitrogen-containing aromatic compounds, pressure-induced irreversible reactions were also reported in pyridine^27–30^, pyrimidine^31^, and s-triazine^31–33^. The required reaction pressure decreased with increasing N/C ratio of the aromatic compounds. A nitrogen-containing carbon nanothread was formed by the slow compression of pyridine up to 23 GPa. The observed N/C ratio of the nanothread was similar to that of the starting pyridine material, indicating that most nitrogen atoms were retained during the polymerization and formation of the sp^3^ bonded carbon^30^. In contrast to PAHs, the behavior of nitrogen-containing aromatic compounds under high pressure and high temperature are hardly investigated. A recent study reported that polymerization of pyridine occurs under high pressure and temperature at approximately 8 GPa and 750–800 K, a reaction temperature lower than that of benzene polymerization^34^. However, details regarding the reaction mechanism and nitrogen behavior during the reaction were not examined.

In this study, the chemistry of a nitrogen-containing aromatic compound under high-pressure and high-temperature conditions was investigated ranging from 0.5 to 1.5 GPa and from 473 to 573 K using a piston-cylinder-type high-pressure apparatus. Naphthyridines (C_8_H_6_N_2_) consist of two condensed aromatic rings as naphthalene, while two of the CH groups have been replaced with nitrogen atoms. There are ten isomers with different positions of nitrogen atoms in the aromatic rings. Here we selected 2,3-naphthyridine as a starting material, in which two nitrogen atoms were localized in one aromatic ring forming a N-N bonding, to investigate the influence of the localized nitrogen atoms on the stability and chemical reactions of the heterocyclic aromatic compound. Precise analysis of the recovered samples was conducted and both relatively light and volatile materials and heavier oligomerized materials were detected to reveal a detailed reaction mechanism.

## Methods

2,3-naphthyridine (Sigma-Aldrich, purity >98%, 15–30 mg) was used as the starting material for the high-pressure and high-temperature experiments. A gold capsule (4 mm outer diameter, 3.6 mm inner diameter) was used as a sample capsule. To remove organic contaminants, the capsule was cleaned with acetone and heated in an oven at 450 °C for 3 h prior to encapsulating the sample.

The sample within the capsule was pressurized using a tungsten carbide piston-cylinder equipped with a hydraulic press. A cylinder with 4 mm inner diameter and 70 mm outer diameter was used without a pressure medium. The detail of the high-pressure apparatus is described in a Supplementary Note. The sample was first compressed to the target pressure at room temperature and subsequently heated by a band-type external heater surrounding the cylinder. The temperature was measured using a K-type thermocouple attached to the top of the cylinder. The compressed sample was recovered from the capsule in distilled dichloromethane to prevent the reaction products from escaping. The solution was initially filtered to remove the sample capsule fragments. No insoluble materials were found in the compressed samples.

The carbon and nitrogen contents of the reaction products were analyzed using an elemental analyzer (EA, Vario EL cube; Elementar Analysensysteme GmbH) after evaporating the solvent. Mass spectra of the reaction products were obtained using a gas chromatography mass spectrometer (GC/MS, JMS-K9; JEOL Co.) equipped with a 30 m × 0.25 mm I.D. capillary column, with a 0.25 μm layer of HP-5 (Agilent Technology Co.). The GC column temperature was programmed as in our previous studies^12,13^. A GC-flame ionization detector (GC/FID, GC-2014; Shimadzu) equipped with an HP-5 capillary column was used for quantitative analysis of the residual 2,3-naphthyridine and reaction products. The GC column temperature was programmed as in the GC/MS measurements. Methyl laurate (C_13_H_26_O_2_), methyl stearate (C_19_H_38_O_2_), and methyl triacontanate (C_31_H_62_O_2_) were used as internal standards for GC/FID and GC/MS analyses. The mass spectra of the reaction products were analyzed using a MALDI-TOF/MS instrument (Ultraflex-III, Bruker), with an α-cyano-4-hydroxycinnamic acid (α-CHCA) matrix. In this study, peaks with >1% of the maximum intensity were considered to be reaction products, similar to the MALDI-TOF/MS study of Chanyshev *et al*.^19^.

## Results and Discussion

**Figure 1:**
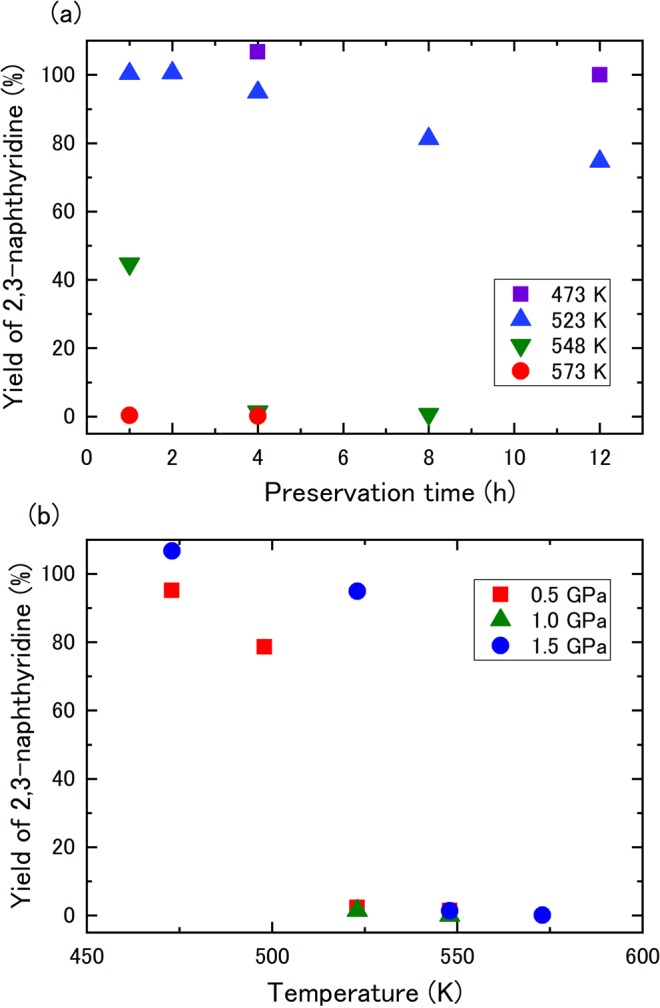
(**a**) Dependence of the residual ratio of 2,3-naphthyridine at 1.5 GPa on preservation time and temperature. (**b**) Dependence of the residual ratio of 2,3-naphthyridine on the pressure and temperature after 4 h heating.

In total, 19 runs were performed at 0.5, 1.0, and 1.5 GPa, changing the temperature from 473 to 573 K with a preservation time of 1–12 h (Table S1). Figure 1a shows the residual ratio of 2,3-naphthyridine at 1.5 GPa as a function of temperature and preservation time, as measured by GC/FID. Most of the 2,3-naphthyridine remained after heating at 473 K and the residual ratio was not significantly changed with preservation time. After heating at 523 K, the residual 2,3-naphthyridine decreased with preservation time, although approximately 75% of the starting material remained after heating for 12 h. In contrast, the residual 2,3-naphthyridine remarkably decreased after heating at 548 K for 4 h to approximately 1%. After heating at 573 K for 1 h, <1% of the 2,3-naphthyridine remained, indicating that the chemical reaction of 2,3-naphthyridine was significant above 548 K at 1.5 GPa. Figure 1b shows temperature dependence of the residual ratio of 2,3-naphthyridine after heating at 0.5–1.5 GPa at a fixed preservation time of 4 h. At 523 K, a significant decrease of 2,3-naphthyridine was observed in the samples at 0.5 and 1.0 GPa while approximately 70% of the 2,3-naphthyridine remained at 1.5 GPa. This suggests that the required reaction temperature increases with increasing pressure.

**Figure 2:**
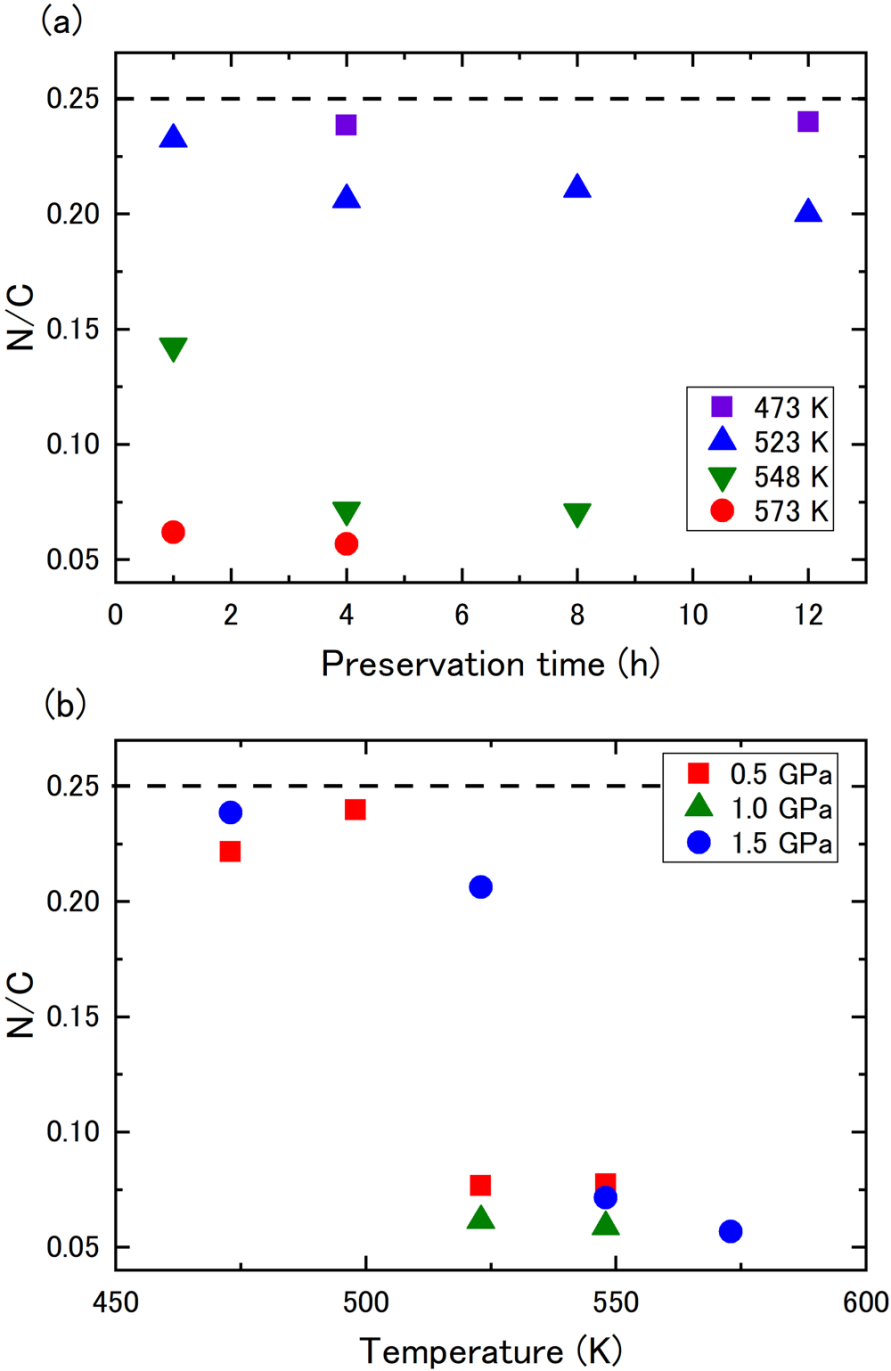
(**a**) Dependence of the N/C ratio of the recovered samples at 1.5 GPa on the preservation time and temperature. (**b**) Dependence of the N/C ratio on the pressure and temperature after 4 h heating. Dotted lines indicate the N/C ratio of 2,3-naphthyridine.

Figure 2a shows the molar ratio of N/C in the recovered samples at 1.5 GPa. After heating at 473 K, the N/C ratio of the recovered sample was 0.240, comparable to that of the starting material (N/C = 0.244) and was unchanged with a preservation time of up to 12 h. At 523 K, the N/C ratio decreased with increasing preservation time from 1 to 4 h, while the N/C ratio remained constant at ∼0.2 between 4 and 12 h. At 548 K, the relative nitrogen content remarkably decreased resulting in an N/C ratio of 0.14 after 1 h of heating, further decreasing to 0.07 at 4 h. After heating at 573 K, the N/C ratio was ∼0.06 after 1 and 4 h. Figure 2b shows the N/C ratios at different pressures with increasing temperature after 4 h of heating. At 0.5 and 1.0 GPa, the N/C ratio significantly decreased after heating above 523 K. The N/C ratio decrease was accompanied by a decreasing residual ratio of 2,3-naphthyridine, suggesting that the chemical reaction involves nitrogen release from the aromatic ring of 2,3-naphthyridine, which is then distributed to the volatile phase(s). When the samples were recovered under ambient conditions and dissolved in dichloromethane solvent, some bubbles were observed. Although the gas phase was not directly analyzed in this study, the bubbles were likely the volatile phase(s) composed of nitrogen and/or ammonia gas. The N/C ratio decreased to approximately 0.05 after the reaction, showing a small amount of nitrogen was left in the recovered samples.

**Figure 3:**
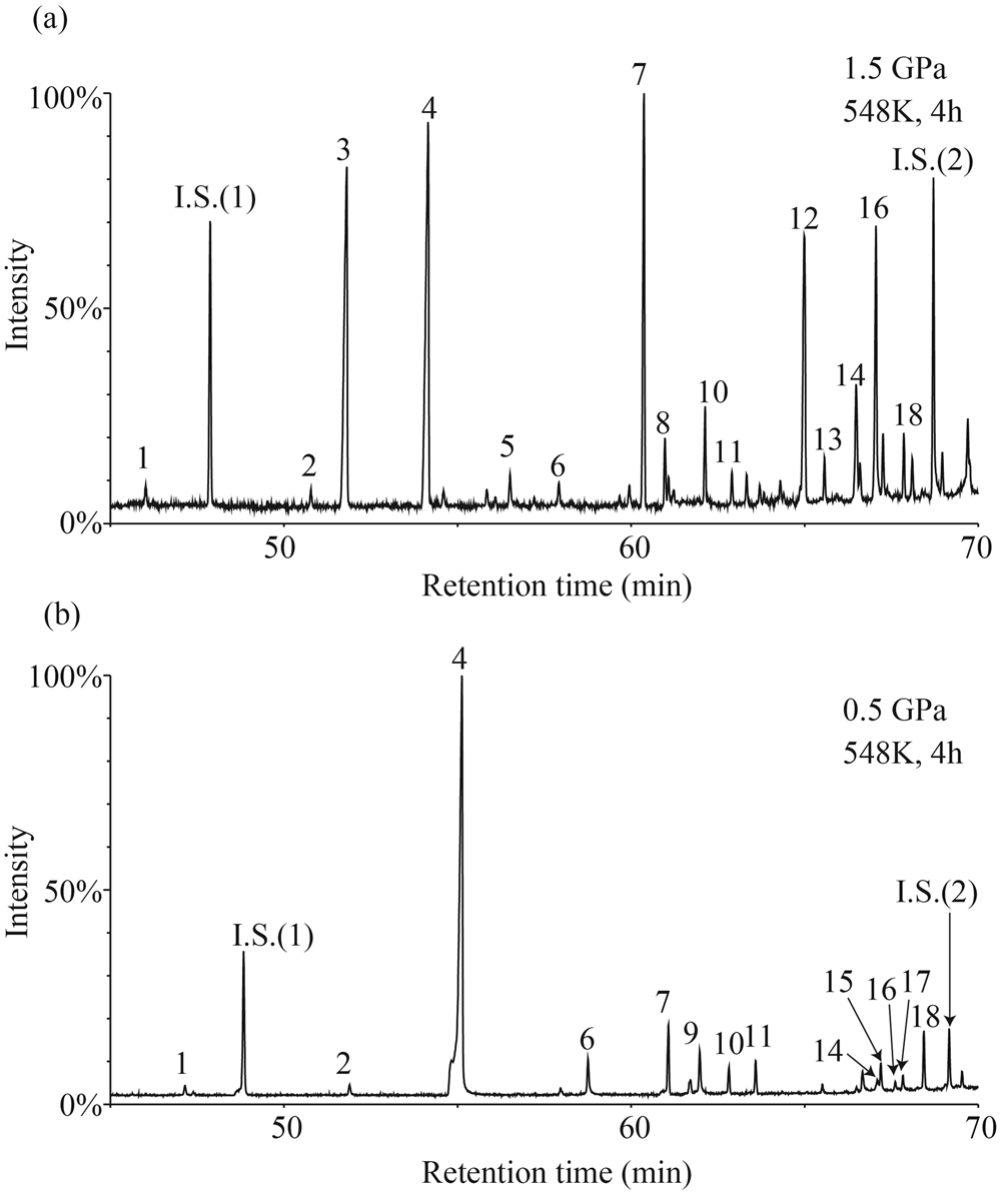
Representative TIC of GC/MS measurements after heating at 548 K for 4 h at (**a**) 0.5 GPa (run no. 5) and (**b**) 1.5 GPa (run no. 16). I.S.(1) and I.S.(2) are the methyl stearate and methyl triacontanoate as internal standards, respectively.

The recovered samples were analyzed using GC/MS to determine the molar mass and possible molecular structures of the reaction products with relatively low masses (*m/z* = 40–500). The products were identified based on comparison of their mass spectra with the database (NIST 02) installed in the GC/MS software. In the samples heated above 523 K regardless of pressure, *o*-xylene (*m/z* = 106) was detected (Fig. S2a). In addition, *o*-tolunitrile (*m/z* = 117) was observed in the samples heated above 523 K at 0.5 GPa and above 548 K at 1.0 and 1.5 GPa (Fig. S2b). The formation of smaller molecules suggests the decomposition of 2,3-naphthyridine to form radicals of *o*-xylene and *o*-tolunitrile. In addition, the total ion chromatogram (TIC) of the samples heated at >523 K at 1.0 and 1.5 GPa, and that of the sample heated at >473 K at 0.5 GPa show peaks corresponding to reaction products with larger *m/z* than that of 2,3-naphthyridine. Figure 3 shows the representative TIC of the reaction products from the samples heated at 548 K at 0.5 and 1.5 GPa for 4 h, with the major products numbered as products 1–18 and listed in Table S1. The identification processes of some of the products are shown in the Supplementary Note and mass spectra of the representative products are shown in Fig. S3.

**Figure 4:**
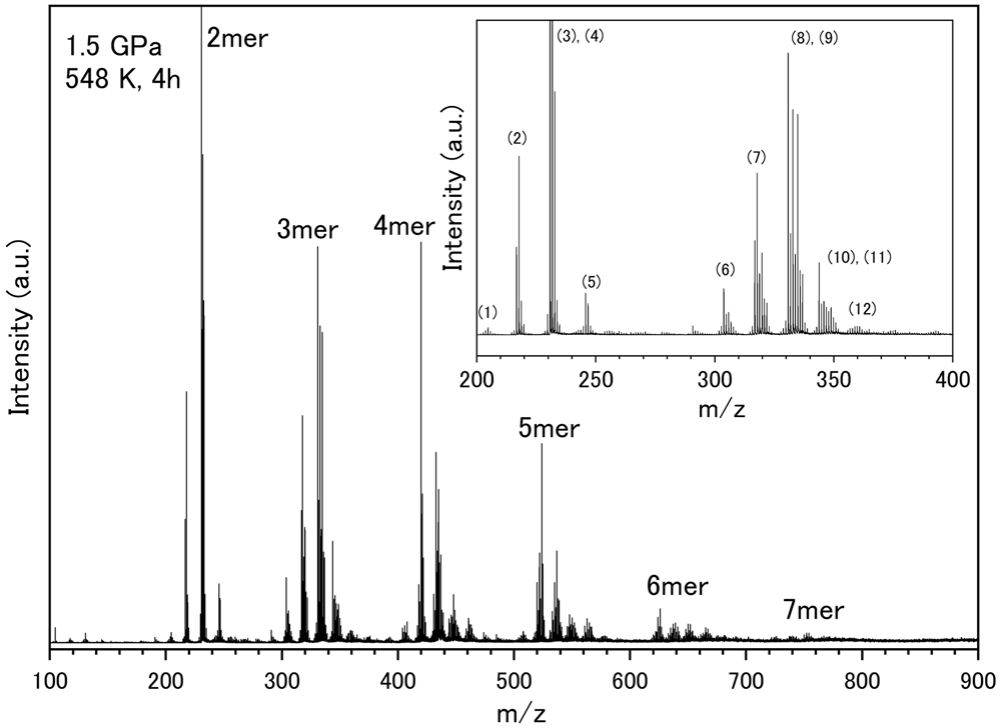
MALDI-TOF/MS spectra of the recovered sample from 1.5 GPa, 548 K, 4 h (run no. 16). The numbers of the inset represent the number of proposed dimerization and trimerization processes as shown in Fig. 5a,b, respectively.

The recovered samples from the 1.5 GPa, 473–573 K and 0.5 GPa, 473–548 K conditions were analyzed using MALDI-TOF/MS to investigate the distribution of product masses with larger mass numbers. Figure 4 shows representative MALDI-TOF/MS spectra of the sample treated at 1.5 GPa, 548 K, and heated for 4 h. Intense peaks were observed at *m/z* = 218, 231, 246, 306, 318, 331, 333, and 335, comparable with the mass numbers of the reaction products observed in the GC/MS analysis. Some of the peaks in the MALDI-TOF spectra were likely [M + H]^+^ ions. The mass pattern is characterized as 5–6 series of peak clusters at intervals of 13–15 mass units. The series of clusters have mass numbers showing periodicities approximately 100 mass units apart, which is comparable to that of the xylene radical. Each cluster is likely to represent dimers, trimers, and heavier oligomers. The largest peak was observed at *m/z* = 780, indicating the formation of oligomers up to 7mers (Fig. 4). Intense peaks of *m/z* = 260 and 261 were additionally detected when the heating temperature was increased to 573 K (Fig. S4a) as well as when the pressure was decreased to 0.5 GPa (Fig. S4b). These mass numbers are consistent with twice the molar weight of 2,3-naphthyridine. However, the exact molecular structure could not be identified because this reaction product was not detected in GC/MS analysis. The result suggests the product was formed from further progress of the chemical reaction at higher temperatures or lower pressures.

**Figure 5:**
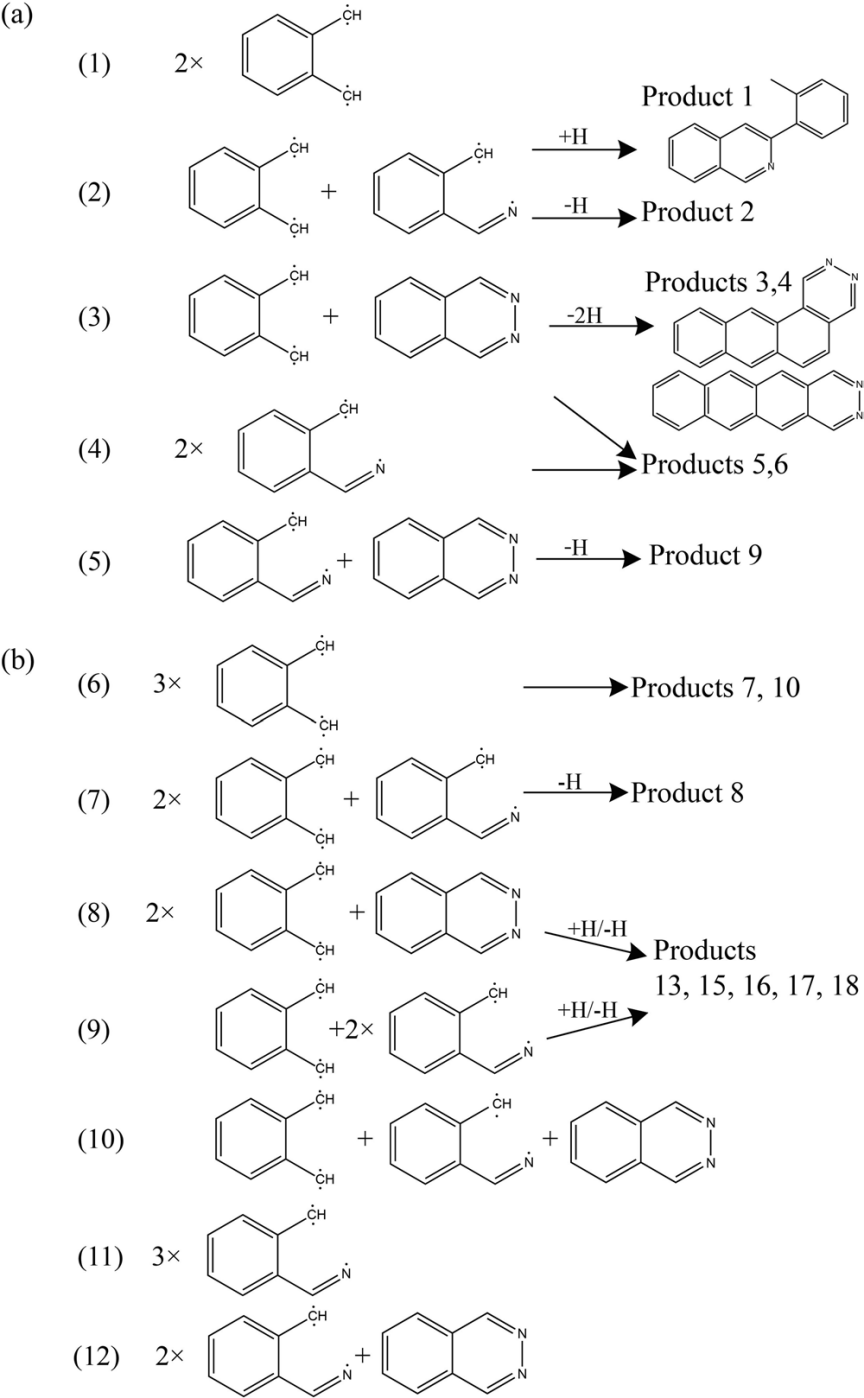
Proposed (**a**) dimerization and (**b**) trimerization process of 2,3-naphthyridine under high pressure and high temperature conditions. The numbers at the left of the molar mass are comparable with the numbers of the inset in Fig. 4. Product numbers are the same as in the GC/MS analysis (Fig. 3, Table S2). A solid line separates the products into dimers and trimers.

From the GC/MS and MALDI-TOF/MS spectra, we propose a major process of dimerization and trimerization in Fig. 5a,b, respectively. Most products are considered to be formed by the oligomerization of *o-*xylene and *o-*tolunitrile radicals with a 2,3-naphthyridine. The representative molar mass of the dimers was 206, 219, 230, 232, and 245. A product with *m/z* = 206 was detected in MALDI-TOF/MS analysis; this was considered to be formed by the dimerization of two *o-*xylene radicals (reaction 1 in Fig. 5a). The product of *m/z* = 219 was detected in both GC/MS (product 1 in Fig. 3) and MALDI-TOF/MS analyses. This product is likely to be formed by the dimerization of *o-*xylene and *o-*tolunitrile radicals with hydrogenation (reaction 2 in Fig. 5a). Product 2 was also formed by the dimerization of *o-*xylene and *o*-tolunitrile radicals with dehydrogenation. Products 3 and 4 were the most abundant products detected in the GC/MS measurements. The possible formation mechanism of these products is the dimerization of an *o*-xylene radical with 2,3-naphthyridine followed by dehydrogenation ((3) in Fig. 5a). The products of *m/z* = 232 (products 5 and 6) were likely to be formed by the dimerization of two *o-*tolunitrile radicals (reaction 4 in Fig. 5a), and the product of *m/z* = 245 (product 9) could be formed by the dimerization of a *o-*tolunitrile radical with 2,3-naphthyridine followed by dehydrogenation. The possible oligomerization mechanisms for product 7–18 are also shown in Fig. 5b as the combinations of *o-*xylene, *o-*tolunitrile radicals, and a 2,3-naphthyridine.

Naphthalene (C_10_H_8_) consists of two condensed aromatic rings as well as 2,3-naphthyridine. Oligomerization of naphthalene has been reported^19,21^, where the reaction temperature (˃800 K) at ambient and high pressures was significantly higher than that of 2,3-naphthyridine. It has been proposed that the oligomerization of naphthalene and other PAHs occur concomitantly with dehydrogenation and without ring opening^19^. In shock experiments of naphthalene at up to 33.7 GPa and 1660 K, dark material comprised of amorphous carbon was predominantly observed with the formation of various methylation, phenylation, and naphthylation products^24^. During the shock experiments of naphthalene, both decomposition and oligomerization occurred, similar to the chemical reaction of 2,3-naphthyridine observed in this study. However, the temperature and pressure of the shock experiments were significantly higher than the values used in this study. The formation of xylene was not reported in the shock experiments of naphthalene. These results indicate that the mechanism of pressure-temperature-induced oligomerization is quite different between naphthalene and 2,3-naphthyridine. The N-N covalent bond in the heterocyclic aromatic compound is easily dissociated and the aromatic ring consisting of both nitrogen and carbon opens at lower temperatures than that of aromatic hydrocarbons. This is similar to a theoretical study that suggested any single covalent bonded compound favors the formation of monomeric molecular N_2_ with a triple bond below 100 GPa^35^. In contrast, cleavage of C-C bonds in the aromatic compounds hardly occurred at the pressure and temperature conditions of this study and the remaining aromatic rings oligomerized to form larger molecules.

**Figure 6:**
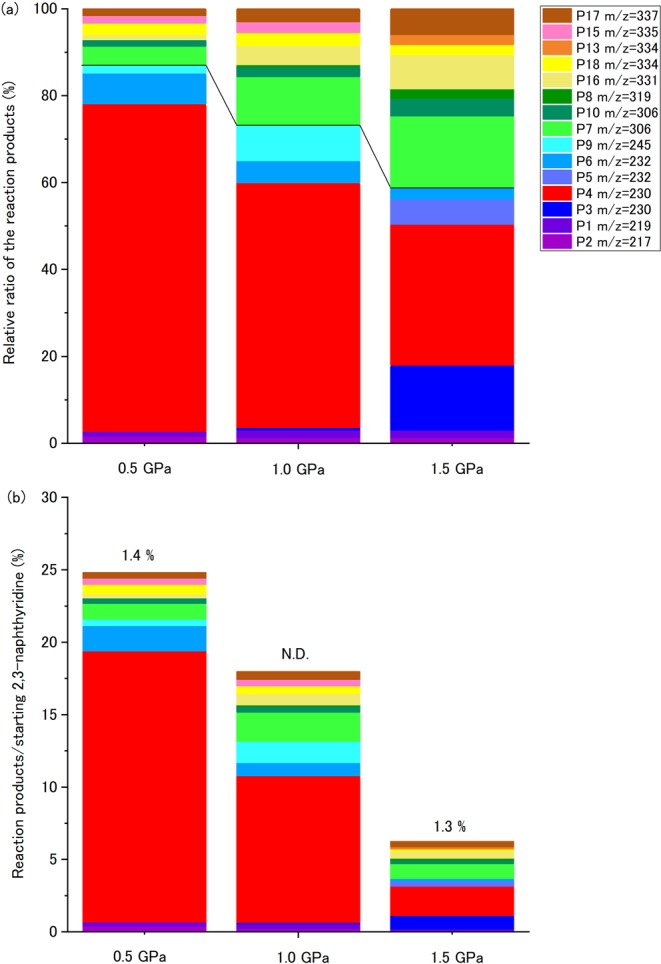
(**a**) Relative molar yield of the reaction products for heating at 548 K for 4 h, detected by GC/FID. A solid line separates the products into dimers and trimers. (**b**) Molar yield of the reaction products. Residual ratio of 2,3-naphthyridine is indicated above each bar. N.D: not detected.

The major decomposition and oligomerization products are similar between 0.5 GPa and 1.5 GPa, although the relative ratio of each product shows the pressure dependence. The molar yields of the major reaction products detected by GC/FID were roughly quantified using the peak area of the chromatogram relative to that of an internal standard (methyl stearate). Because suitable standards for products were not available, to quantify the reaction products we used the response factor of benz[*a*]anthracene, which shows similar structure to those of the major products (product 3 and 4 in Fig. 3). Figure 6a shows the ratios of the relative reaction products between 0.5 and 1.5 GPa, at 548 K. The products could be classified as dimers (*m/z* = 217–245) and trimers (*m/z* = 306–337). At 0.5 GPa, over 80% of the quantified products are dimers, while the abundance falls to approximately 60% at 1.5 GPa. The relative ratio of the trimers increases with increasing pressure suggesting the trimers preferentially form at higher pressure. The molar yields of the major reaction products are shown in Fig. 6b. The total yield of the detected reaction products decreases with increasing pressure, even though the residual ratio of 2,3-naphthyridine is quite similar between the recovered samples. The result suggests that undetected products in GC/MS are likely the heavier products, as observed in MALDI-TOF/MS analysis, and were preferentially formed at higher pressures. The results suggest heavier oligomers prefer to form with increasing pressure. Similar pressure dependence of oligomerization was also reported by a theoretical study of methane oligomerization under high-pressure and high-temperature conditions, in which heavier reaction products preferred to form at higher pressure, although both pressure and temperature were significantly higher than the present study^36^. Intermolecular distances shorten with increasing pressure and this could induce a formation of heavier oligomers at higher pressure.

## Conclusion

Chemical reactions of 2,3-naphthyridine were investigated at 0.5–1.5 GPa and 473–573 K using a piston-cylinder-type high-pressure apparatus. A distinct decrease in the residual 2,3-naphthyridine was observed in GC/FID analysis of the samples recovered from >523 K at 0.5/1.0 GPa and >548 K at 1.5 GPa. The recovered samples exhibited lower N/C ratios than in the original material in addition to forming *o-*xylene and *o*-tolunitrile products, as detected by GC/MS analysis. These results suggest that the decomposition of the aromatic ring occurred with nitrogen release. Various heavy oligomers (up to approximately *m/z* = 780) were detected by GC/MS and MALDI/TOF-MS analyses. Most oligomers observed in GC/MS analysis contained aromatic ring(s) in their structure and the MALDI/TOF-MS spectra of the recovered samples contained products with mass numbers showing periodicities of approximately *m/z* = 100. This indicated that oligomerization occurred up to 7mers between *o*-xylene, *o*-tolunitrile, and the residual 2,3-naphthyridine. At higher temperatures, peaks with a mass approximately twice of that of 2,3-naphthyridine were observed, implying simple dimerization of 2,3-naphthyridine as the second step of the chemical reaction. The major reaction mechanism was similar at 0.5–1.5 GPa, although the required reaction temperature increased with increasing pressure. Quantification of the representative dimers and trimers indicated that larger products preferentially formed in the samples at higher pressure, suggesting that oligomerization is favored at higher pressure.

## Supplementary information

Supplementary Information

## References

[1] Ehrenfreund P, Charnley SB (2000). Organic molecules in the interstellar medium, comets, and meteorites: A voyage from dark clouds to the early earth. Annu. Rev. Astron. Astrophys..

[2] Tielens, A. In *Annu*. *Rev*. *Astron*. *Astrophys*. *Vol*. *46 Annual Review of Astronomy and Astrophysics*, 289–337 (Annual Reviews, 2008).

[3] Waite JH (2009). Liquid water on Enceladus from observations of ammonia and Ar-40 in the plume. Nature.

[4] Bezard B (2009). Composition and chemistry of Titan’s stratosphere. Philos. Trans. R. Soc. A-Math. Phys. Eng. Sci..

[5] Becker L, Glavin DP, Bada JL (1997). Polycyclic aromatic hydrocarbons (PAHs) in Antarctic Martian meteorites, carbonaceous chondrites, and polar ice. Geochim. Cosmochim. Acta.

[6] Sephton MA (2002). Organic compounds in carbonaceous meteorites. Nat. Prod. Rep..

[7] Thiery MM, Leger JM (1988). High-pressure solid-phase of benzene. 1. Raman and X-ray studies of C6H6 at 294 K up to 25 GPa. J. Chem. Phys..

[8] Pruzan P (1990). Transformation of benzene to a polymer after static pressurization to 30 GPa. J. Chem. Phys..

[9] Cansell F, Fabre D, Petitet JP (1993). Phase-transitions and chemical-transitions of benzene up to 550 C and 30 GPa. J. Chem. Phys..

[10] Ciabini L, Santoro M, Bini R, Schettino V (2002). High pressure reactivity of solid benzene probed by infrared spectroscopy. J. Chem. Phys..

[11] Ciabini L (2007). Triggering dynamics of the high-pressure benzene amorphization. Nat. Mat..

[12] Shinozaki A (2014). Pressure-induced oligomerization of benzene at room temperature as a precursory reaction of amorphization. J. Chem. Phys..

[13] Shinozaki A (2016). Stability and partial oligomerization of naphthalene under high pressure at room temperature. Chem. Phys. Lett..

[14] O’Bannon E, Williams Q (2016). Vibrational spectra of four polycyclic aromatic hydrocarbons under high pressure: implications for stabilities of PAHs during accretion. Phys. Chem. Miner..

[15] Root S, Gupta YM (2009). Chemical Changes in Liquid Benzene Multiply Shock Compressed to 25 GPa. J. Phys. Chem. A.

[16] Chen B (2015). Linearly Polymerized Benzene Arrays As Intermediates, Tracing Pathways to Carbon Nanothreads. J. Am. Chem. Soc..

[17] Fitzgibbons TC (2015). Benzene-derived carbon nanothreads. Nat. Mat..

[18] Li X (2017). Mechanochemical Synthesis of Carbon Nanothread Single Crystals. J. Am. Chem. Soc..

[19] Chanyshev AD, Litasov KD, Furukawa Y, Kokh KA, Shatskiy AF (2017). Temperature-induced oligomerization of polycyclic aromatic hydrocarbons at ambient and high pressures. Sci Rep.

[20] Chanyshev AD, Litasov KD, Shatskiy AF, Furukawa J, Ohtani E (2014). Stability Conditions of Polycyclic Aromatic Hydrocarbons at High Pressures and Temperatures. Geochem. Inter..

[21] Chanyshev AD (2015). Oligomerization and carbonization of polycyclic aromatic hydrocarbons at high pressure and temperature. Carbon.

[22] Davydov VA (2004). Conversion of polycyclic aromatic hydrocarbons to graphite and diamond at high pressures. Carbon.

[23] Chanyshev AD (2017). Transition from melting to carbonization of naphthalene, anthracene, pyrene and coronene at high pressure. Phys. Earth Planet. Inter..

[24] Mimura K (2004). Shock-induced pyrolysis of naphthalene and related polycyclic aromatic hydrocarbons (anthracene, pyrene, and fluoranthene) at pressures of 12-33.7 GPa. J. Anal. Appl. Pyrolysis.

[25] Mimura K, Nishida T (2017). Hydrogen and Hydrocarbon Gases, Polycyclic Aromatic Hydrocarbons, and Amorphous Carbon Produced by Multiple Shock Compression of Liquid Benzene up to 27.4 GPa. J. Phys. Chem. A.

[26] Guo LH, Wang GL, Yan ZE, Zhang X (2016). Mechanism for covalent dimerization of pyridine: 4 + 2 dimerization, an MP2 investigation. Chem. Phys. Lett..

[27] Zhuravlev KK (2010). Raman and infrared spectroscopy of pyridine under high pressure. Phys. Rev. B.

[28] Fanetti S, Citroni M, Bini R (2011). Structure and reactivity of pyridine crystal under pressure. J. Chem. Phys..

[29] Yasuzuka T, Komatsu K, Kagi H (2011). A Revisit to High-pressure Transitions of Pyridine: A New Phase Transition at 5 GPa and Formation of a Crystalline Phase over 20 GPa. Chem. Lett..

[30] Li X (2018). Carbon Nitride Nanothread Crystals Derived from Pyridine. Ame. Chem. Soc..

[31] Li SR (2014). Effect of pressure on heterocyclic compounds: Pyrimidine and s-triazine. J. Chem. Phys..

[32] Citroni M, Fanetti S, Bini R (2014). Pressure and Laser-Induced Reactivity in Crystalline s-Triazine. Jour. Phys. Chem. C.

[33] Citroni M (2015). Structural and Electronic Competing Mechanisms in the Formation of Amorphous Carbon Nitride by Compressing s-Triazine. J. Phys. Chem. C.

[34] Kondrin MV (2017). Bulk graphanes synthesized from benzene and pyridine. Crystengcomm.

[35] Brazhkin VV, Nikolaev NA, Shulga YM, Lebedc YB, Kondrin MV (2018). The structure and synthesis of organic crystalline polymers: hints from ab initio computation. Crystengcomm.

[36] Spanu L, Donadio D, Hohl D, Schwegler E, Galli G (2011). Stability of hydrocarbons at deep Earth pressures and temperatures. Proc. Natl. Acad. Sci. USA.

