# Supplementary material for: Decomposition and oligomerization of 2,3-naphthyridine under high-pressure and high-temperature conditions
Source: Sci Rep. 2019 May 14;9:7335. doi: 10.1038/s41598-019-43868-2 (PMC6517384; doi:10.1038/s41598-019-43868-2)
Supplement: Supplementary file 1 — Supplementary Information [file 41598_2019_43868_MOESM1_ESM.pdf]

Decomposition and oligomerization of 2,3-naphthyridine under high-pressure and high-temperature conditions

Ayako Shinozaki[a*], Koichi Mimura[b], Tamihito Nishida[b]

[a]Faculty of Science, Hokkaido University, N10 W8, Kita-ku, Sapporo, Hokkaido 060-0810, Japan

[b]Department of Earth and Planetary Sciences, Graduate School of Environmental Studies, Nagoya University, Nagoya 464-8601, Japan

*Corresponding author: Ayako Shinozaki.

Tel.: +81-(0)11-706-2727 fax: +81-(0)11-746-0394

E-mail: shinozaki.aya@sci.hokudai.ac.jp

Supplementary Methods

Piston-cylinder type high-pressure and high-temperature apparatus

The sample within the capsule was pressurized using a tungsten carbide piston-cylinder equipped with a hydraulic press. The relationship between press load and applied pressure was determined in advance by monitoring the phase transitions of a standard material (NH_4F), which exhibits two phase transitions at 0.36 and 1.17 GPa[1]. The phase transitions as a function of the applied load were determined by recording the ram strokes, which reflect the decreased sample volume with increasing pressure. Discontinuous change of the stroke indicates a phase transition (Fig. S1). At the heating by the band heater, the temperature fluctuated within ±5 K during the experiments. Sample pressure increased within 0.1 GPa at 523 K and 0.2 GPa at 573 K. Approximately 15 min was required to reach 573 K after turning on the heater. After turning off the heater, the sample cooled to room temperature within 50 min and was then decompressed to ambient pressure.

Supplementary notes

GC/MS measurements

Figure S3a shows the mass spectrum of product 1 in the TIC (Fig. 3), which is quite similar to that of 1-benzylisoquinoline ($C_{16}H_{13}N$) in the NIST 02 database. Considering the molecular structure of *o*-xylene and *o*-tolunitrile, the product of dimerization would likely be an isomer of 1-benzylisoquinoline, such as 3-(2-methylphenyl)isoquinoline. Figure S3b shows the mass spectrum of the most intense peak in the TIC, peak 4 (Fig. 3), which has m/z = 230. The mass spectrum of peak 3 is quite similar to that of peak 4, indicating that these products are isomers. Compared to the NIST 02 database, the mass spectrum of benzo-phenazine ($C_{16}H_{10}N_2$) is similar to those of peaks 3 and 4. Considering the molecular structure of 2,3-naphthyridine, the products can be assigned as isomers of naphtho-2,3-naphthyridine. The molar masses of the major products are listed in Table S2 and were determined from molecular ion peaks in the mass spectra. The other products detected in the chromatogram could not be identified, because the database for these molecules was not available. Most mass spectra showed typical aromatic compound features, as shown in Fig. S3c and S3d.

Supplementary Figure S1

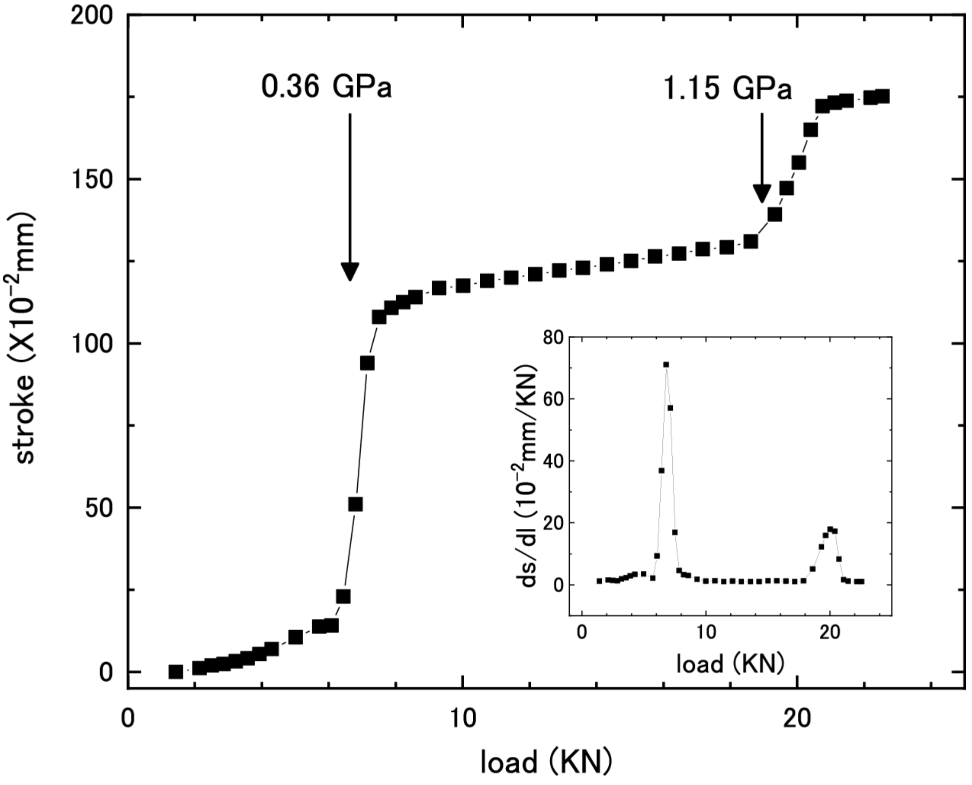

Figure S1 Dependence of the applied load on the stroke of the ram when ammonium fluoride is filled in a gold capsule. Inset: Dependence of the applied load on the derivative of the stroke by load.

Supplementary Figure S2

(a)

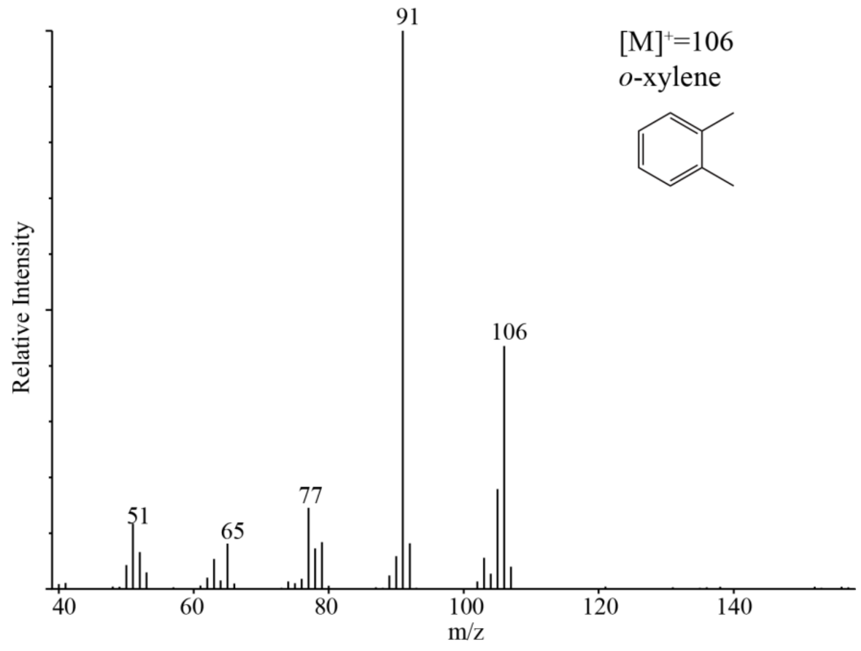

(b)

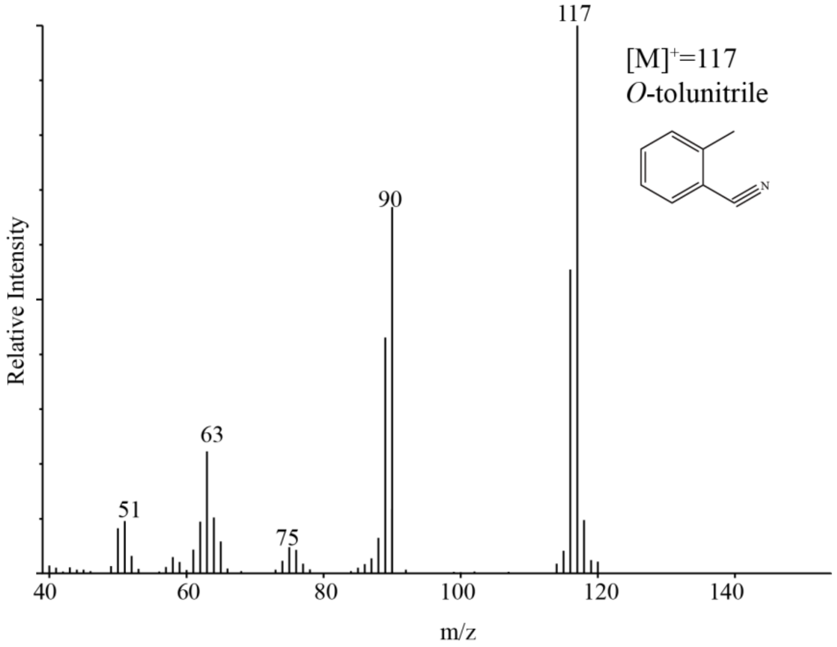

Figure S2 (a) Mass spectra of *o*-xylene (m/z = 106) and (b) *o*-tolunitrile (m/z = 117) from the sample obtained at 0.5 GPa, 548 K, 4 h (run no. 5).

Supplementary Figure S3

(a)

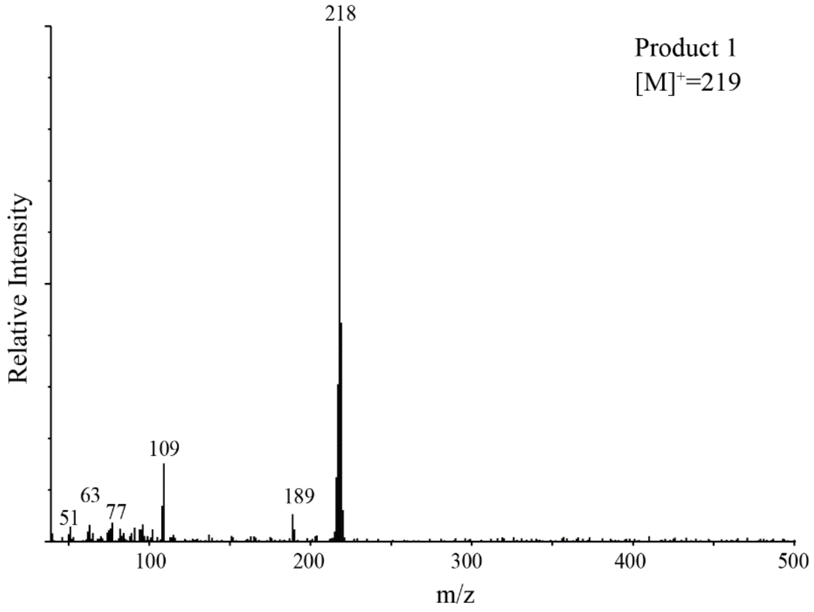

(b)

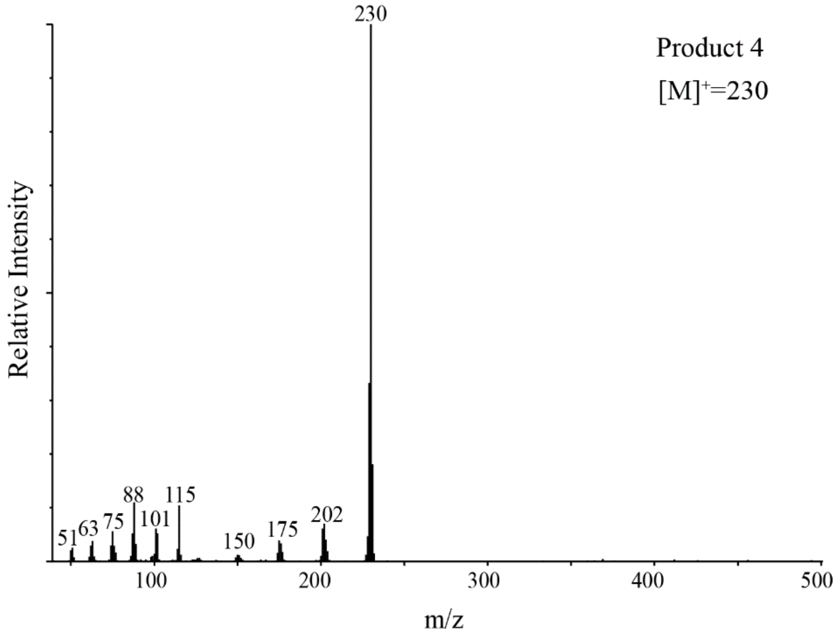

(c)

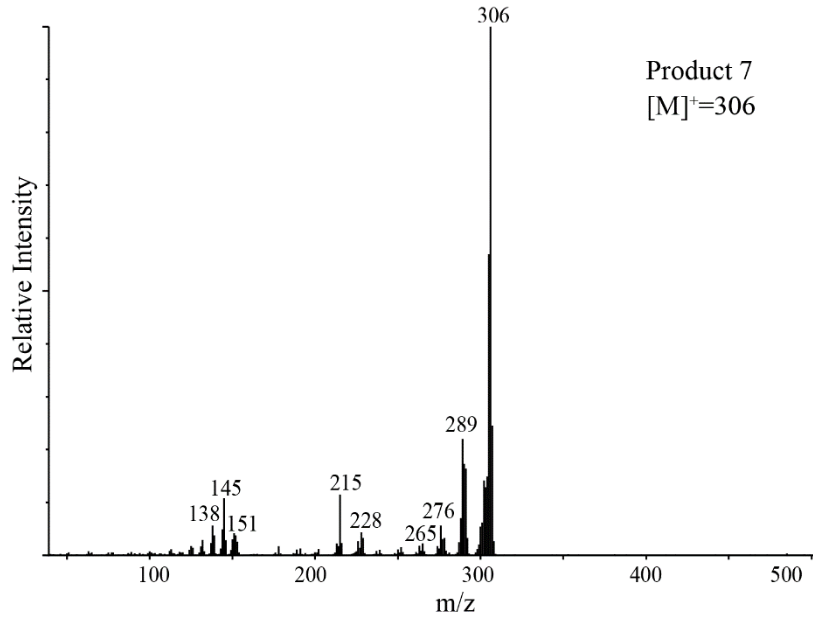

(d)

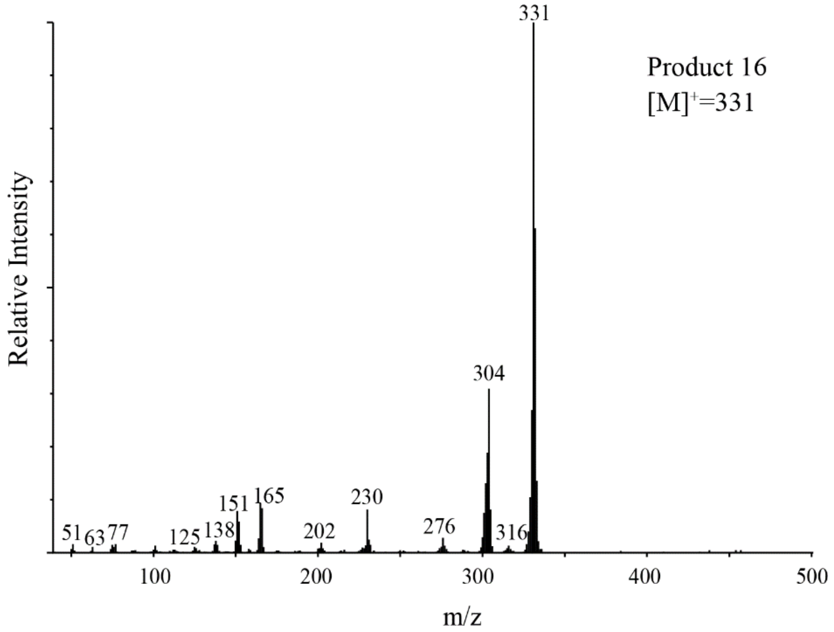

Figure S3 Representative mass spectrum of (a) product 1 (b) product 4, (c) product 7, and (d) product 16 of the sample obtained at 1.5 GPa, 548 K, 4 h (run no. 16).

Supplementary Figure S4

(a)

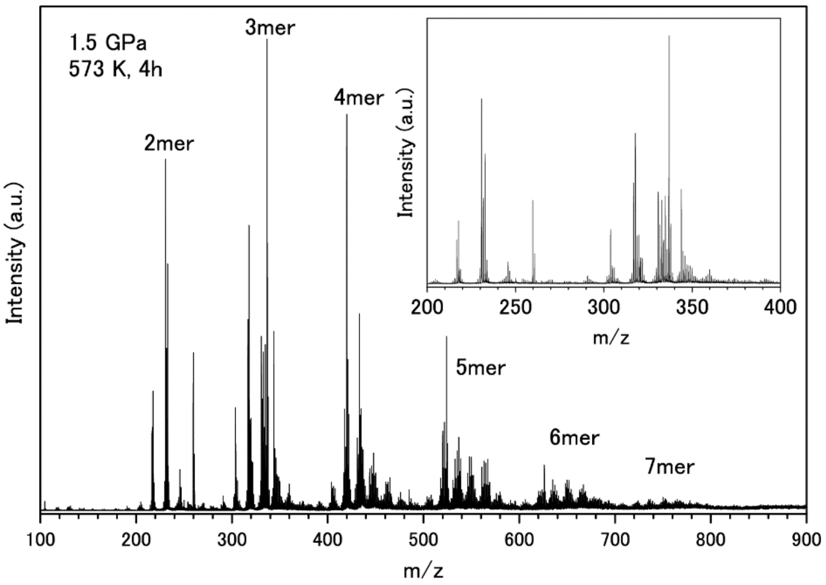

(b)

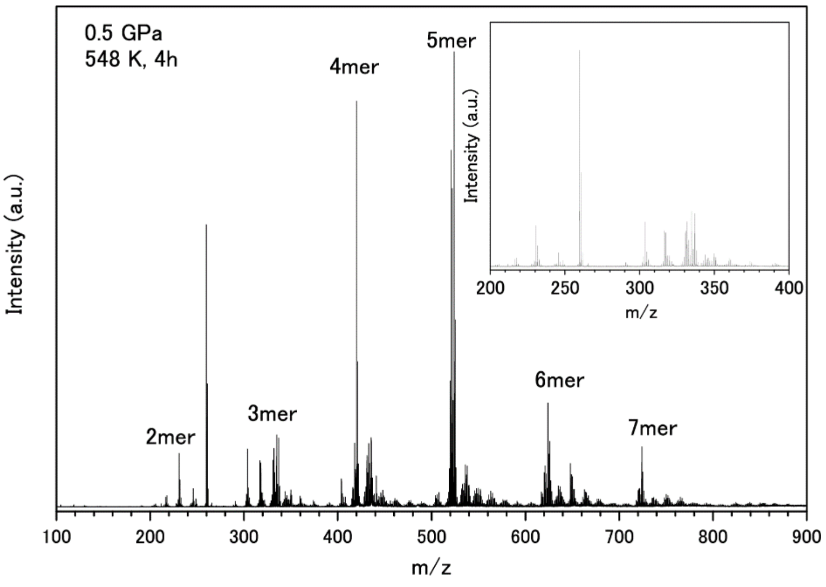

Figure S4 Representative MALDI-TOF/MS spectra of the recovered samples. (a) 1.5 GPa, 573 K, 4 h (run no. 19), and (b) 0.5 GPa, 548 K, 4 h (run no. 5).

Supplementary Table S1 Experimental conditions of the present study.

Run no.	Pressure (GPa)	Temperature (K)	Preservation time (h)	Remaining 2,3-naphthyridine (%)	N/C
1	0.5	293	4	92.2	0.24
2	0.5	473	4	95.2	0.22
3	0.5	498	4	78.6	0.24
4	0.5	523	4	2.3	0.08
5	0.5	548	4	1.4	0.08
6	1.0	523	4	1.4	0.06
7	1.0	548	4	-	0.06
8	1.5	473	4	106.7	0.24
9	1.5	473	12	100.0	0.24
10	1.5	523	1	100.3	0.23
11	1.5	523	2	100.6	N
12	1.5	523	4	94.9	0.21
13	1.5	523	8	81.3	0.21
14	1.5	523	12	74.7	0.20
15	1.5	548	1	44.7	0.14
16	1.5	548	4	1.3	0.07
17	1.5	548	8	0.6	0.07
18	1.5	573	1	0.4	0.06
19	1.5	573	4	0.1	0.05

+: detected, -: not detected, N: not measured

Supplementary Table S2 Molar mass of the major products in representative runs (%).

	m/z	Run 2	Run 4	Run 5	Run 6	Run 7	Run 8	Run 12	Run 16	Run 19
[a]P,T		0.5 GPa, 473 K	0.5 GPa 523 K	0.5 GPa 548 K	1.0 GPa 523 K	1.0 GPa 548 K	1.5 GPa 473 K	1.5 GPa 523 K	1.5 GPa 548 K	1.5 GPa 573 K
o-xylene	106	-	+	+	+	+	-	+	+	+
o-tolunitrile	117	-	+	+	-	+	-	-	+	+
Product 1	219	-	0.42	0.28	0.34	0.31	-	-	0.10	0.10
Product 2	217	-	0.35	0.39	0.00	0.24	-	-	0.08	0.11
Product 3	230	-	0.24	-	0.63	0.09	0.04	0.30	0.94	0.27
Product 4	230	1.08	19.44	18.72	7.71	10.13	0.03	0.22	2.03	1.98
Product 5	232	-	-	-	-	-	-	-	0.36	0.03
Product 6	232	-	0.84	1.75	0.47	0.91	-	-	0.17	0.57
Product 7	306	-	1.79	1.09	3.16	2.00	-	-	1.03	0.99
Product 8	319	-	0.24	-	-	-	-	-	0.14	0.12

Product 9	245	-	0.71	0.44	0.57	1.47	-	-	-	-
Product 10	306	-	0.42	0.37	0.27	0.42	-	-	0.25	0.20
Product 11	292	-	0.62	0.51	0.43	0.73	-	+	0.09	-
Products 12	231+334	-	1.10	-	2.88	0.16	0.04	0.29	1.27	-
Product 13	334	-	0.38	-	0.23	0.00	-	-	0.14	0.16
Products 14	317+334	-	1.95	0.35	2.36	0.65	-	-	0.61	0.34
Product 15	335	-	0.71	0.43	0.49	0.45	0.03	0.11	-	0.15
Product 16	332	-	0.71	0.28	0.72	0.79	0.03	0.12	0.49	0.22
Product 17	337	-	0.71	0.40	0.39	0.54	0.03	0.14	0.37	0.37
Product 18	334	-	0.50	0.66	0.38	0.53	-	-	0.15	0.18

a: The duration time is 4h

+: detected, -: not detected

Reference

1 Taniguchi, H. *et al.* Short piston-cylinder pressure cells based on Ni-Cr-Al cylinders and their application to fragile materials. *Rev. Sci. Instrum.* **81**, 033903, doi:10.1063/1.3310197 (2010).
